# Prevalence of influenza A and B and respiratory syncytial virus infections before and during COVID-19 pandemic in the pediatric population in Lebanon: A retrospective study

**DOI:** 10.1371/journal.pone.0325001

**Published:** 2025-06-06

**Authors:** Reem Eid, Alain Sayad, Wadana Samaan, Pascale Salameh, Roula Antoine Farah

**Affiliations:** 1 Department of Pediatrics, Lebanese American University Medical Center - Rizk Hospital, Beirut, Lebanon; 2 Department of Pediatric Gastroenterology, Lebanese American University Medical Center - Rizk Hospital, Beirut, Lebanon; 3 Department of Pediatrics, Lebanese American University Medical Center - Rizk Hospital, Beirut, Lebanon; 4 Gilbert and Rose-Mary Chagoury School of Medicine, Lebanese American University Medical Center, Byblos, Lebanon; 5 University of Nicosia Medical School, Nicosia, Cyprus; 6 Faculty of Pharmacy, Lebanese University, Hadat, Lebanon; 7 Institut National de Santé Publique, Epidémiologie Clinique et Toxicologie (INSPECT-LB), Dekwaneh, Metn, Lebanon; 8 Department of Pediatric Hematology and Oncology, Lebanese American University Medical Center - Rizk Hospital, Beirut, Lebanon; Lagos State University, NIGERIA

## Abstract

Seasonal influenza and RSV outbreaks cause considerable morbidity and mortality in the pediatric population worldwide. The COVID-19 pandemic has changed virus epidemiology. Until today, it is still unclear how this pandemic affected the transmission of common respiratory viruses. The present study aimed at comparing the prevalence of RSV and influenza A/B infection before and during the COVID-19 pandemic in the Lebanese pediatric population. A multicenter retrospective cross-sectional study was performed from September 2018 to December 2022 at the Lebanese American University Medical Center – Rizk Hospital and Abou Jaoude Hospital in Lebanon. Included were children (0–18 years) tested for Influenza A and B and RSV by Rapid Influenza Diagnostic Test and Rapid Antigen Testing, respectively, taken by nasopharyngeal swab at both hospitals where the study was conducted. Data collection was retrieved from the medical records of the patients. The statistical analysis was performed using SPSS software, version 30.0. This study has considered all ethical measures. Among a total of 1069 children tested, 19.7% tested positive for influenza A, 11.9% for influenza B, and 13.8% for RSV. The study found that young infants were significantly less susceptible to contracting these viruses compared to older children and adolescents (p < 0.001). A statistically significant difference in the odds of testing positive was observed between the two hospitals (p = 0.011), and a significant temporal trend in influenza circulation was noted (p < 0.05). The prevalence of co-infection was low with no statistically significant differences (p = 0.779). The COVID-19 pandemic and its associated preventive measures led to a significant decrease in the spread of influenza A and B and RSV among the Lebanese pediatric population. Conversely, the post-lockdown period saw a notable resurgence of these infections, with low coinfection occurrences. These results have important implications for public health strategies aimed at controlling respiratory virus infections.

## Introduction

Worldwide, seasonal outbreaks of influenza and respiratory syncytial virus (RSV) have a substantial negative influence on young children’s health, including the pediatric population and newborns, leading to high rates of morbidity and mortality [1]. Acute respiratory infection symptoms account for a significant proportion of primary care visits and hospital admissions throughout the winter months [2]. Children aged under one-year-old have the highest incidence of RSV infections and hospitalization, and these rates tend usually to increase in the winter season [3,4]. Therefore, an estimated one hundred million cases and over eight hundred hospitalizations in children under five years are caused by seasonal influenza viruses each year worldwide [5].

During the COVID-19 pandemic, these viruses’ epidemiology has changed significantly. The year 2020 has shown a notable decline in the spread of RSV and influenza A and B, mostly due to the lockdowns, social distancing, and closure of public places, as well as all the associated preventive measures imposed by the World Health Organization (WHO), the Centers of Disease Control and Prevention (CDC) and the governments worldwide [6]. Whereas, as the public health and social measures were gradually lifted, several countries, including Australia, South Africa, New Zealand, France, Spain, the United States, and Japan, experienced a seasonality shift and delayed RSV and influenza outbreaks, resulting in a greater number of infected patients [7,8]. Remarkably, there was a notable resurgence of RSV infections and influenza cases during the cold season of 2021/2022, with epidemiological studies conducted in different countries showing peaks in the number of cases that were higher than those documented in pre-pandemic studies [9–11].

Notably, uncertainty surrounds the full effect of the COVID-19 pandemic and related mitigating measures on the spread of common respiratory viruses and possible interactions and connections between them [12,13]. Therefore, some studies in the literature showed that there exist coinfections between COVID-19 and these respiratory viruses, while others didn’t find any interaction between those viruses [12,14]. For instance, Steponaviciene et al. (2023) emphasized in the results of their study that the frequency of coinfection between COVID-19, influenza, and RSV viruses in the pediatric population is low (7.2%) [15]. Similarly, Yan et al. (2023) showed a coinfection prevalence equal to 2.45% among patients diagnosed with COVID-19 infection [16]. Conversely, Cong et al. (2022) found no significant association between RSV or influenza infections and COVID-19 infection [17].

The first case of Covid-19 in Lebanon was reported on the 20th of February 2020 and the WHO declared the pandemic over on May 5, 2023. In Lebanon, a retrospective study conducted by Fakih et al. (2024) aiming to compare the prevalence and characteristics of Respiratory Tract Infections (RTIs) before and during the COVID-19 pandemic, among hospitalized Lebanese children, showed a notable increase in RTI’ prevalence during the pandemic when compared to the pre-pandemic period [18]. However, this study didn’t specifically focus on RSV and influenza A and B among children, which raises concerns about the necessity of investigating the prevalence of these infections before and during the COVID-19 pandemic among the pediatric population in Lebanon.

In other words, the present study aimed at comparing the prevalence of RSV and influenza A and B infection before and during the COVID-19 pandemic in the pediatric population in Lebanon.

## Materials and methods

### Study design

A multicenter retrospective cross-sectional study was performed from September 2018 to December 2022 at the Lebanese American University Medical Center – Rizk Hospital (LAUMC-RH) and Abou Jaoude Hospital in Lebanon.

### Population and sample size

The population of this study included all children tested for influenza A and B and RSV by Rapid Influenza Diagnostic Test (RIDT) and Rapid Antigen Testing (RAT), respectively, taken by nasopharyngeal swabs at both hospitals where the study was conducted. The inclusion criteria were as follows: patients aged under 18 years old, tested for these viruses either in pediatric outpatient settings or in pediatric wards and emergency rooms, during the time frame of the study. The analysis was performed based on the results of the laboratory of these hospitals, which were included in the medical records of the patients. The testing for other viruses was not taken into consideration. There were no exclusion criteria to mention.

### Data collection

Data collection was performed between January 2023 and June 2023 using a pre-set data collection form and the required information was retrieved from the medical records of the patients diagnosed between September 2018 to December 2022. The data collection form included the demographic information of the patients, noting their gender, age, and the medical center they were admitted to. It also included information regarding testing for Influenza A (FluA), Influenza B (FluB), and RSV, as well as the date of the testing and the results. An additional analysis was conducted to detect coinfections between Influenza A and B, Influenza A and RSV, Influenza B and RSV and resulting outcomes.

### Statistical analysis

The statistical analysis was performed using Statistical Package for the Social Science (SPSS) software, version 28. First, a descriptive analysis was performed where categorical data was presented as frequencies and percentages. Second, a bivariate analysis was performed to identify statistically significant correlations between the variables, using the Chi-square test or the Fisher exact test in case of expected values lower than 5. Finally, a binary logistic regression was done to identify predictors of RSV, influenza A, and influenza B, using the ENTER method and a confirmed non-significant Hosmer-Lemeshow test for sampling adequacy. P-values inferior to 0.05 were considered statistically significant throughout the study.

### Ethical considerations

This study was approved by the Institutional Review Board (IRB) and the Institutional Ethical Committee (IEC) of the LAUMC-RH and Abou Jaoudeh Hospital (*IRB#: LAUMCRH.RF2.22/Feb/2022)*. It was conducted in accordance with the principles of the World Medical Association Helsinki Declaration as well as the local law. The names and information of the participants in this study were not shared with third parties and were fully anonymized prior to the statistical analysis. Participants were designated by codes to conceal their name and identity. Additionally, as the study design was retrospective and there was no contact with the patients or their parents, the IRB waived the need to obtain written or verbal informed consent from the families.

## Results

### Demographic characteristics

This study included 1069 pediatric patients aged between 0–18 years, receiving care at the LAUMC-RH (n = 597, 55.8%) and Abou Jaoude Hospital (n = 472, 44.2%) in Lebanon. The population sample was distributed between 55.9% of males (n = 598) and 44.1% of females (n = 471). The age distribution is categorized into four groups, showcasing a significant concentration of patients in the 1–10 years’ age group, which represents 59.6% (n = 637) of the study population. The 11–18 years’ age group accounts for 22.6% (n = 242), followed by 1–12 months with 15.2% (n = 163), and the 0–1-month group, being the smallest, constitutes 2.5% (n = 27) (Table 1).

**Table 1:** Demographic Characteristics of the Study Population (N = 1069).

		Frequency	Percent
**Gender**	Female	471	44.1
	Male	598	55.9
**Age**	0-1month	27	2.5
	1-12 months	163	15.2
	1-10 years	637	59.6
	11-18 years	242	22.6
**Medical Center**	LAUMC-RH	597	55.8
	Abou Jaoudeh	472	44.2

### Influenza testing

The findings (Table 2), shedding light on the incidence rates and seasonal variations of influenza infections across a span of over four years, revealed that 19.7% of the tests returned positive for Influenza A, and 11.9% returned positive for Influenza B. The rarity of co-infections, with only 0.5% of cases testing positive for both Influenza A and B, points to the exceptional nature of such occurrences.

**Table 2:** Influenza Test Results and Dates of Testing (N = 973).

		Frequency	Percent
**Influenza Test**	FluA+	192	19.7
	FluB+	116	11.9
	Both Negative	660	67.8
	Both Positive (Coinfection)	5	0.5
**Date of Influenza Test**	Sep 2018-Sep 2019	203	20.9
	Sep 2019-Sep 2020	375	38.5
	Sep 2020-Sep 2021	13	1.3
	Sep 2021-Sep 2022	83	8.5
	Sep 2022-Dec 2022	299	30.7

The temporal distribution of influenza tests conducted from September 2018 to December 2022 reveals significant fluctuations in testing activity. The initial period from September 2018 to September 2019 accounted for 20.9% of the tests, setting the baseline for the study’s scope. A notable increase in testing to 38.5% of the total occurred in the following year. However, the period from September 2020 to September 2021 marked a dramatic decrease to merely 1.3% of the tests, possibly influenced by the global COVID-19 pandemic and its subsequent effects on healthcare resources and public health priorities. Testing rates gradually recovered, with the final quarter of 2022 witnessing a surge in tests, accounting for 30.7% of the total, indicating a renewed focus on monitoring influenza activity among the pediatric population (Table 2).

Table 3 shows the analysis of influenza test outcomes, segregated by gender, age, medical center, and test period. The analysis across different age groups reveals statistically significant differences in influenza infection rates (P < 0.001). The youngest age group (0–1 month) shows a notably lower incidence of FluA+ but a higher proportion of FluB+ infections. Contrastingly, the 11–18 years’ group exhibits the highest proportion of FluA+ infections (29.1%) and a moderate level of FluB+ infections (14.5%), indicating age-specific trends in influenza susceptibility and highlighting the importance of targeted vaccination and prevention strategies.

**Table 3:** Influenza test in function of demographic characteristics.

		Influenza Test				P.value
		FluA+	FluB+	Both Negative	Both Positive (Coinfection)	
**Gender**	Female	86	55	293	3	0.845
		19.7%	12.6%	67.0%	0.7%	
	Male	106	61	367	2	
		19.8%	11.4%	68.5%	0.4%	
**Age**	0-1month	1	3	11	0	**<0.001**
		6.7%	20.0%	73.3%	0.0%	
	1-12 months	3	4	112	0	
		2.5%	3.4%	94.1%	0.0%	
	1-10 years	120	75	405	5	
		19.8%	12.4%	66.9%	0.8%	
	11-18 years	68	34	132	0	
		29.1%	14.5%	56.4%	0.0%	
**Medical Center**	Rizk	113	41	400	0	**<0.001**
		20.4%	7.4%	72.2%	0.0%	
	Abou Jaoudeh	79	75	260	5	
		18.9%	17.9%	62.1%	1.2%	
**Date of Influenza Test**	Sep 2018-Sep 2019	28	13	162	0	**<0.001**
		13.80%	6.40%	79.80%	0.00%	
	Sep 2019-Sep 2020	76	87	211	1	
		20.30%	23.20%	56.30%	0.30%	
	Sep 2020-Sep 2021	0	4	9	0	
		0.00%	30.80%	69.20%	0.00%	
	Sep 2021-Sep 2022	7	1	75	0	
		8.40%	1.20%	90.40%	0.00%	
	Sep 2022-Dec 2022	81	11	203	4	
		27.10%	3.70%	67.90%	1.30%	

A significant difference in influenza test results between the two medical centers is observed (P < 0.001), with LAUMC-RH showing a higher prevalence of FluA+ cases (20.4%) compared to Abou Jaoude Hospital (18.9%). Additionally, Abou Jaoude Hospital has a slightly higher rate of co-infections (1.2%).

The date of the influenza test reveals significant fluctuations in influenza positivity rates over time (P < 0.001), with a marked increase in FluA+ and FluB+ cases from September 2022 to December 2022. The period of September 2020 to September 2021, corresponding to the peak months of the COVID-19 pandemic, shows a drastic reduction in FluB+ cases.

The logistic regression analysis between influenza results and other characteristics, provided in Table 4, showed a significant variation in influenza positivity across different age groups (Wald = 32.446, df = 3, P < 0.001). Specifically, children aged 0–1 month have significantly lower odds (Exp(B) = 0.188) of testing positive for influenza compared to the reference group (p = 0.021). Additionally, the medical center where care is received was shown to be a significant predictor of influenza test results (B = 0.399 for Abou Jaoudeh versus LAU-Medical Center Rizk Hospital, P-value = 0.011). The period of the influenza test was also shown to be a significant predictor of test results (Wald = 37.408, df = 4, P < 0.001), with significant variability across different testing periods. The period from September 2018 to September 2019 shows a significant increase in the odds of testing positive (Exp(B) = 2.72, P < 0.001), indicating a higher risk during this timeframe. Therefore, the period from September 2021 to September 2022 shows increased odds of influenza positivity (Exp(B) = 1.838, P = 0.008), suggesting temporal trends in influenza circulation.

**Table 4:** Logistic regression for influenza results predictors.

	B	S.E.	Wald	df	P.value	Exp(B) =ORa	95% C.I.for EXP(B)	
							Lower	Upper
**Gender (M vs F)**	−0.049	0.146	0.113	1	0.737	0.952	0.716	1.267
***Age Group***			32.446	3	<0.001			
0-1month	−1.672	0.724	5.333	1	**0.021**	0.188	0.045	0.776
1-12 months	0.146	0.614	0.057	1	0.812	1.157	0.348	3.852
1-10 years	0.62	0.623	0.99	1	0.320	1.858	0.548	6.299
**Medical Center (Abou Jaoudeh vs LAUMCRH Hospital)**	0.399	0.157	6.442	1	**0.011**	1.49	1.095	2.027
***Date of Influenza Test***			37.408	4	<0.001			
Sep 2018-Sep 2019	1.001	0.21	22.736	1	**<0.001**	2.720	1.803	4.105
Sep 2019-Sep 2020	0.505	0.637	0.627	1	0.428	1.656	0.475	5.774
Sep 2020-Sep 2021	−0.745	0.42	3.147	1	0.076	0.475	0.209	1.081
Sep 2021-Sep 2022	0.609	0.23	7.016	1	**0.008**	1.838	1.171	2.883
**Constant**	−1.616	0.64	6.383	1	0.012	0.199		

a. Variable(s) entered in the model: Gender (reference:Female), Age Group (reference: 11–18 years), Medical Center (reference: LAUMC-Rizk Hospital), Date of Influenza Test (reference “Sep 2022-Dec 2022”).

### RSV test

The analysis of RSV test outcomes for the study period (Table 5) reveals that out of 268 tests conducted, 37 were positive, accounting for 13.8% of the total. The temporal analysis of RSV testing from September 2018 to December 2022 shows fluctuations in testing frequencies, which could reflect seasonal variations, changes in testing guidelines, or the impact of public health initiatives. The period “September 2018 - September 2019” saw the highest number of RSV tests (n = 88, 32.8%), potentially indicating a heightened focus on RSV surveillance or an increase in respiratory illnesses prompting testing during this time. Between September 2019 and September 2020, the frequency of tests slightly decreased to 58 tests (21.6%). Between September 2020 and September 2021, a significant drop in testing was shown to 13 tests (4.9%). In the period between September 2021 and September 2022, the testing frequencies began to increase with 55 tests (20.5%) and remain stable during the period between September 2022 and December 2022 with 54 tests (20.1%).

**Table 5:** RSV test results (N = 268).

		Frequency	Percent
**RSV Test**	Positive	37	13.8
	Negative	231	86.2
**Date of RSV Test**	Sep 2018-Sep 2019	88	32.8
	Sep 2019-Sep 2020	58	21.6
	Sep 2020-Sep 2021	13	4.9
	Sep 2021-Sep 2022	55	20.5
	Sep 2022-Dec 2022	54	20.1

Table 6 shows the analysis of RSV test outcomes, segregated by gender, age, medical center, and test period. The distribution of RSV test results by gender indicates a slight variance in positivity rates, with females showing a positivity rate of 15.4% and males at 12.6%, with no statistically significant difference (P = 0.593).

**Table 6:** RSV test results in function of demographics.

		RSV Test		P.value
		Positive	Negative	
**Gender**	Female	18	99	0.593
		15.4%	84.6%	
	Male	19	132	
		12.6%	87.4%	
**Age**	0-1month	9	12	**<0.001**
		42.9%	57.1%	
	1-12 months	20	88	
		18.5%	81.5%	
	1-10 years	7	108	
		6.1%	93.9%	
	11-18 years	1	23	
		4.2%	95.8%	
**Medical Center**	Rizk	28	171	0.845
		14.1%	85.9%	
	Abou Jaoudeh	9	60	
		13.0%	87.0%	
**Date of RSV Test**	Sep 2018-Sep 2019	13	75	0.904
		14.8%	85.2%	
	Sep 2019-Sep 2020	9	49	
		15.5%	84.5%	
	Sep 2020-Sep 2021	1	12	
		7.7%	92.3%	
	Sep 2021-Sep 2022	6	49	
		10.9%	89.1%	
	Sep 2022-Dec 2022	8	46	
		14.8%	85.2%	

A clear age-related trend is observed in RSV positivity rates (P < 0.001). Infants aged 0–1 month have the highest positivity rate at 42.9%, followed by 1–12 months at 18.5%. The positivity rate notably decreases in older age groups, with 1–10 years at 6.1% and 11–18 years at the lowest rate of 4.2%. The positivity rates for RSV tests at LAUMC-RH and Abou Jaoudeh medical centers are 14.1% and 13.0%, respectively, with no significant statistical difference between the two locations (P = 0.845). Similarly, the analysis didn’t reveal any significant year-over-year differences in RSV positivity rates in RVS test results (P = 0.904).

The logistic regression analysis between RSV results and other characteristics, provided in Table 4, showed that gender is not a predictor of testing positive for RSV (P = 0.244). Whereas, the analysis across age groups indicates clear differences in the likelihood of RSV positivity among the different age categories (P < 0.001) with a Wald statistic of 21.538. Specifically, infants aged 0–1 month (Exp(B) = 0.266) are significantly less likely to test positive for RSV compared to the reference category, with their odds reducing by approximately 73.4%. The likelihood further decreases for the 1–12 months age group (Exp(B) = 0.071). Children aged 1–10 years exhibit the lowest odds of RSV positivity (Exp(B) = 0.04), indicating a substantial decrease in risk compared to the baseline group.

Conversely, the results showed that the medical center attended does not significantly impact the odds of testing positive for RSV within this population (P = 0.685). Similarly, the temporal analysis across different testing periods does not reveal significant differences in RSV positivity rates (P = 0.551) (Table 7).

**Table 7:** Logistic regression for RSV results predictors.

	B	S.E.	Wald	df	P.value	Exp(B)	95% C.I.for EXP(B)	
							Lower	Upper
Gender (M vs F)	−0.451	0.387	1.358	1	0.244	0.637	0.298	1.361
*Age Group*			21.538	3	**<0.001**			
0-1month	−1.323	0.523	6.393	1	**0.011**	0.266	0.096	0.743
1-12 months	−2.638	0.611	18.666	1	**<0.001**	0.071	0.022	0.237
1-10 years	−3.211	1.149	7.807	1	0.005	0.04	0.004	0.383
Medical Center (Abou Jaoudeh vs LAUMCRH Hospital)	−0.184	0.454	0.164	1	0.685	0.832	0.341	2.026
*Date of RSV Test*			3.042	4	0.551			
Sep 2018-Sep 2019	0.347	0.51	0.463	1	0.496	1.415	0.521	3.84
Sep 2019-Sep 2020	−0.706	1.119	0.398	1	0.528	0.494	0.055	4.424
Sep 2020-Sep 2021	−0.671	0.578	1.348	1	0.246	0.511	0.165	1.587
Sep 2021-Sep 2022	0.02	0.527	0.001	1	0.970	1.02	0.363	2.868
Constant	0.216	0.61	0.125	1	0.724	1.241		

a. Variable(s) entered in the model: Gender (reference: female), Age Group (reference: 11–18 years), Medical Center (reference: LAUMC-Rizk Hospital), Date of RSV Test (reference “Sep 2022-Dec 2022”).

### Coinfections

The analysis of existing coinfections between RSV test results and influenza test outcomes (FluA and FluB) showed there is no statistically significant association between the presence of influenza and RSV infections (P = 0.779). Out of 172 total tests, only 1 case (0.6%) tested positive for RSV among those who also tested positive for FluA, no cases (0.0%) tested positive for RSV among those who tested positive for FluB (Table 8).

**Table 8:** Influenza and RSV test results between 2018 and 2023.

		RSV Test		Total	P.value
		Positive	Negative		
**Influenza Test**	**FluA+**	1	11	12	0.779
		0.6%	6.4%	7.0%	
	**FluB+**	0	2	2	
		0.0%	1.2%	1.2%	
	**Both Negative**	23	135	158	
		13.4%	78.5%	91.9%	
**Total**		24	148	172	
		14.0%	86.0%	100.0%	

## Discussion

Amidst the COVID-19 pandemic, the literature showed a significant drop in the spread of respiratory diseases due to the implementation of infection control measures aimed at reducing the strain on healthcare systems [6,19]. Despite lasting over three years as a worldwide health emergency, the COVID-19 pandemic witnessed a gradual easing of infection control measures where individuals were back again to their normal daily life [20]. As a consequence, the burden of respiratory infections resurfaced globally [9]– [11]. The present study examined the epidemiological shifts in Influenza A/B and RSV, as acute respiratory infections, among the pediatric Lebanese population.

Among a total of 1069 children tested for either Influenza A/B or RSV in the two hospitals where the study was conducted, the results showed that 19.7% of the children tested positive for influenza A, 11.9% tested positive for influenza B, and 13.8% tested positive for RSV in the period of the study. Moreover, the temporal distribution of tests varied in the period ranging between September 2018 and December 2022, as the results showed that the baseline testing prevalence (20.9% for influenza A and B and 32.8% for RSV) has notably decreased during the era of COVID-19 pandemic and yet it gradually increased in the timeframe between September 2021 and September 2022. These results highlight the effect of the preventive measures during COVID-19 pandemic on the reduction of the spread and transmission of other respiratory viruses, noting influenza and RSV. In addition, these results might indicate a reduction in healthcare visits during the pandemic as individuals avoided healthcare settings to minimize their risk and their children’s risks of contracting the COVID-19. This avoidance could have led to fewer children being tested for respiratory viruses during this timeframe. Moreover, the lifting of preventive measures could have led to a resurgence in healthcare visits and testing for respiratory symptoms, which might explain the observed increase in testing for influenza and RSV from September 2021 to September 2022. In other words, the gradual increase in testing indicates that as normal activities resumed, so did the circulation of common respiratory viruses, leading to more children exhibiting symptoms and subsequently being tested. These results are concordant with those mentioned in the literature, where various studies conducted in different contexts showed a decrease in testing for respiratory viruses, especially during the peak of COVID-19 pandemic. A notable example is the study of Groves et al. (2021), conducted in Canada, showing a significant decrease in the prevalence of respiratory infections during the pandemic [21], and the study of Principi et al. (2023) highlighting the resurgence of non-SARS-Cov-2 infections after lifting COVID-19 preventive measures [22].

Remarkably, the results showed that young infants were significantly less susceptible to contract influenza or RSV viruses than older children and adolescents (p < 0.001). These results might be due to the fact that young infants have more protective maternal antibodies, transferred through the placenta or breastfeeding, and are less prone to be exposed to external environments and social interactions compared to older children. Conversely, older individuals might have had previous exposures or vaccinations against these respiratory viruses, and this might influence the higher detection rates of infections [23]. Remarkably, these results seem to be aligned with those mentioned in the study of Korsten et al. (2022), showcasing the higher prevalence of acute respiratory infections among children of preschool age and older [23].

Moreover, the statistically significant difference between the odds of testing positive in both hospitals (p = 0.011) underscores the importance of taking into account the healthcare setting as a factor that influences the transmission and detection of respiratory viruses, notably the influenza. In this context, the higher odds of positivity at one medical center suggest that specific factors related to the healthcare environment, patient population, and healthcare practices, might play a role. Similar results were as well mentioned in the study of Mulpuru et al. (2015) [24].

Nonetheless, the results of the present study showed significant temporal trend in influenza circulation (p < 0.05), heavily influenced by seasonal variations and the impact of the COVID-19 pandemic and its related public health measures. For instance, the implementation and subsequent ease of public health measures to control COVID-19 has significant impact on influenza transmission. Hence, the periods with reduced influenza positivity – September 2019 to September 2021, align with the stringent COVID-19 measures, while the increase in September 2021 to September 2022 corresponds with the easing of these measures. Therefore, during the height of the COVID-19 pandemic, people might have been less likely to seek medical care for mild respiratory symptoms among their children due to fear of contracting COVID-19 in healthcare settings or due to the overwhelming focus on COVID-19 virus, leaving aside other potential respiratory infections. Subsequently, this might had led to fewer influenza tests and lower reported positivity rates during this period.

Furthermore, the prevalence of coinfection between influenza A and B was 0.5%, and it was 0.6% between RSV and influenza A, whereas there was no coinfection between RSV and influenza B. Moreover, these coinfections weren’t shown to have statistically significant differences (p = 0.779). These results indicate that it is uncommon for pediatric patients to be simultaneously infected with multiple respiratory viruses. This refers to the concept of “virus interference” known as the inhibition of infection or replication of another virus in the same host if one is already infected [25]. These results seem to be concordant with those mentioned in the study of Kim et al. (2022) highlighting that coinfection with influenza and another respiratory virus is relatively rare and did not show significant clinical differences compared to single infections [26].

Additional limitations to this study could be evoked: the study suggests that the stringent COVID-19 measures led to a decrease in influenza positivity rate, but does not fully explore the long-term effects of these measures on respiratory virus transmission post-pandemic, leaving a gap in understanding how these changes may persist or evolve. The results may be influenced by confounding factors not accounted for in the analysis. The lack of detailed demographic and clinical data could limit the interpretation of the findings. The choice of two hospitals may also lead to a selection bias. Lastly, using Rapid Influenza Diagnostic Test (RIDT) and Rapid Antigen Testing (RAT) may limit study findings accuracy, reflecting a potential information bias. Further studies that take into account these limitations are suggested to confirm our findings.

## Conclusion

The COVID-19 pandemic and its associated preventive measures significantly reduced the spread of influenza A, influenza B, and RSV among the Lebanese pediatric population. However, the post-lockdown period saw a notable resurgence of these infections, with low rates of coinfection occurrences. The study also identified significant age-related differences in infection rates as well as significant differences in influenza positivity rates between the two medical centers included in this study. Additionally, the study provides valuable epidemiological data by comparing before and during pandemic. These results have important public health implications for controlling respiratory virus infections. Hence, continuous surveillance and research are still essential to prevent and control respiratory infections in the future.

## Supporting information

S1 Fig Number of influenza tests performed between 2018 and 2022 by year. (TIF)

S2 Fig Number of RSV tests performed between 2018 and 2022 by year. (TIF)
