# Supplementary figures and images for: Prevalence of influenza A and B and respiratory syncytial virus infections before and during COVID-19 pandemic in the pediatric population in Lebanon: A retrospective study
Source: PLoS One. 2025 Jun 6;20(6):e0325001. doi: 10.1371/journal.pone.0325001 (PMC12143508; doi:10.1371/journal.pone.0325001)

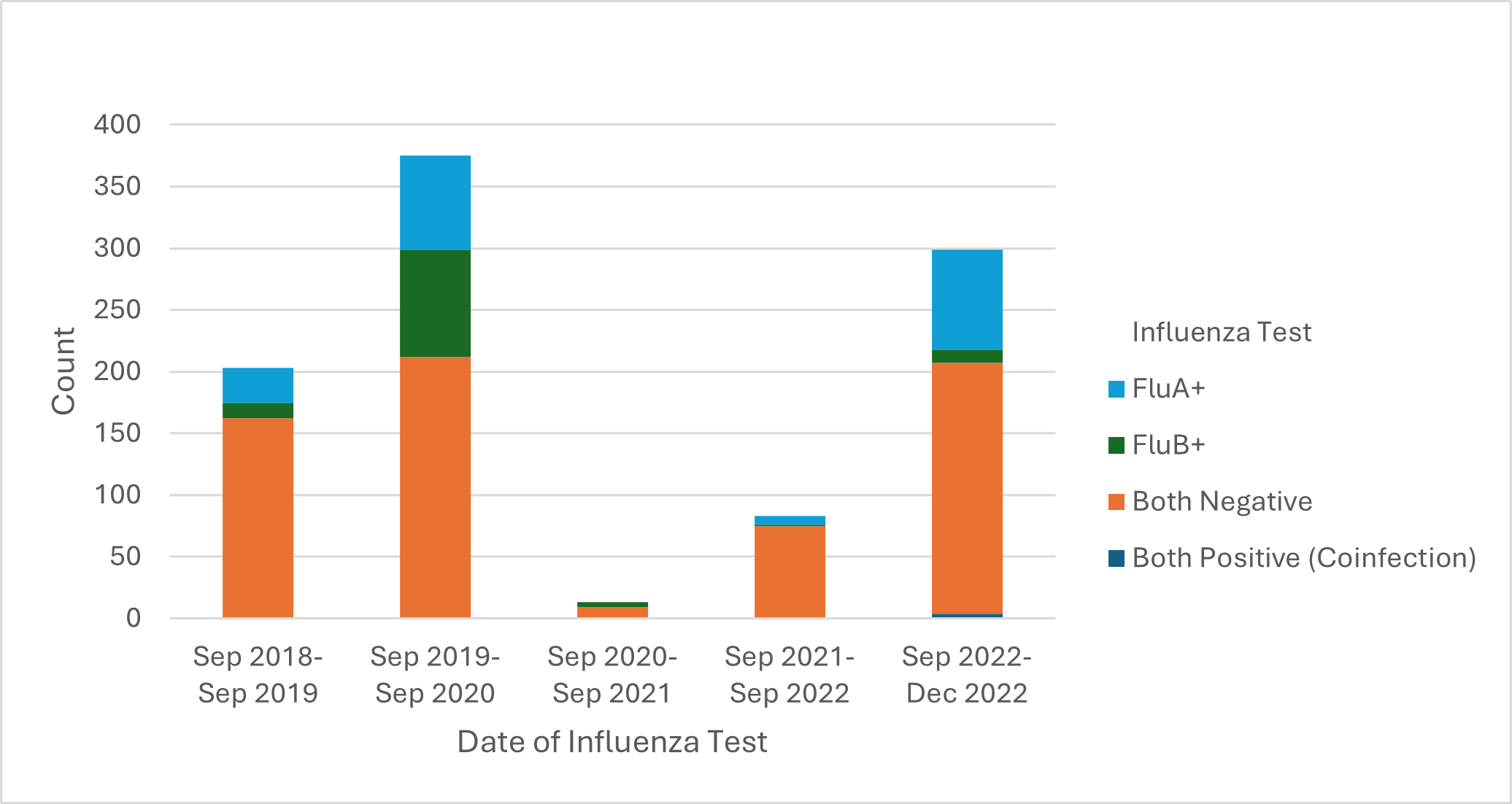

Supplement: S1 Fig — (TIF) [file pone.0325001.s001.tif]

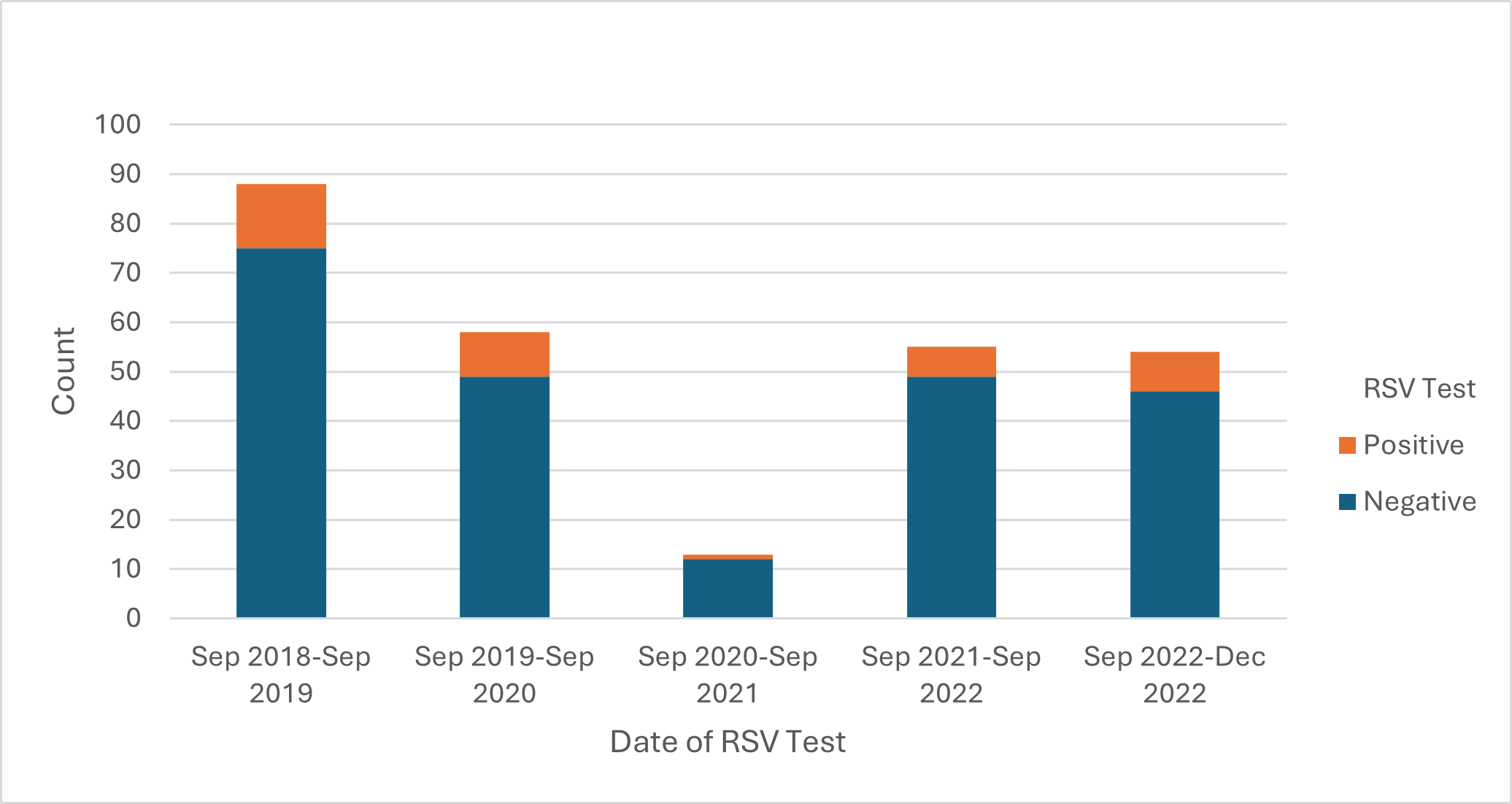

Supplement: S2 Fig — (TIF) [file pone.0325001.s002.tif]
